# The Effects of Raspberry Consumption on Glycemic Control and Inflammation Markers in Adults: A Systematic Review and Meta-Analysis of Randomized Controlled Trials

**DOI:** 10.1016/j.cdnut.2024.102161

**Published:** 2024-05-03

**Authors:** Mostafa Shahraki Jazinaki, Mina Nosrati, Mahla Chambari, Tannaz Jamialahmadi, Amirhossein Sahebkar

**Affiliations:** 1 Student Research Committee, Mashhad University of Medical Sciences, Mashhad, Iran; 2 Department of Nutrition, Faculty of Medicine, Mashhad University of Medical Sciences, Mashhad, Iran; 3 International UNESCO Center for Health-Related Basic Sciences and Human Nutrition, Mashhad University of Medical Sciences, Mashhad, Iran; 4 Noncommunicable Diseases Research Center, Neyshabur University of Medical Sciences, Neyshabur, Iran; 5 Pharmaceutical Research Center, Pharmaceutical Technology Institute, Mashhad University of Medical Sciences, Mashhad, Iran; 6 Medical Toxicology Research Center, Mashhad University of Medical Sciences, Mashhad, Iran; 7 Biotechnology Research Center, Pharmaceutical Technology Institute, Mashhad University of Medical Sciences, Mashhad, Iran; 8 Applied Biomedical Research Center, Mashhad University of Medical Sciences, Mashhad, Iran

**Keywords:** raspberry, glycemic control, inflammation markers, systematic review, meta-analysis

## Abstract

Despite observing the health benefits of raspberry consumption in some recent studies, there is still no consensus regarding this effectiveness on inflammatory markers and glycemic control. This study aimed to investigate this effectiveness by performing a meta-analysis. The PubMed, Web of Science, and Scopus databases were comprehensively searched until December 2023 to find relevant randomized controlled trials. Eligible studies were screened, and relevant information was extracted. The overall effect size of raspberry consumption on each of the outcomes was estimated by following the random-effects model in the form of a 95% confidence interval (CI) and a weighted mean difference (WMD). Raspberry consumption led to a significant increase in insulin concentrations (WMD: 1.89 μU/mL; 95%CI: 1.45, 2.34; *P* < 0.001) and a significant decrease in tumor necrosis factor-α (TNF-α) concentrations (WMD: −3.07 pg/mL; 95%CI: −5.17, −0.97; *P* = 0.004), compared with the control groups. Raspberry consumption did not have a significant effect on fasting blood glucose, insulin, hemoglobin A1C, glucose tolerance tests, homeostatic model assessment for insulin resistance, C-reactive protein, and interleukin-6 concentrations. This review revealed that raspberry consumption led to a significant increase and decrease in insulin and TNF-α concentrations, respectively. However, to draw a more accurate conclusion, it is necessary to conduct studies with a larger sample size in the future.

The current study's protocol has been registered in the PROSPERO system as CRD42023477559.

## Introduction

Recent decades have seen an abundance of research exploring the pharmacologic effects of medicinal plants, dietary factors, and herb-derived natural products in combating cardiometabolic disorders [[1], [2], [3], [4], [5], [6], [7], [8]]. Among these dietary agents are raspberries. Raspberries belong to the Rosaceae family, which grows widely in East Asian countries [9]. This plant is rich in flavonoids, tannins, phenolic acids, organic acids, tyrosol, and resveratrol, which have been associated with potential anti-inflammatory effects and improved glycemic control in several studies [[10], [11], [12]]. Increased concentrations of postprandial glucose, as well as elevated concentrations of biomarkers of oxidative stress and inflammation, have been associated with cardiovascular disease (CVD) [13,14]. Consequently, understanding the impacts of raspberry consumption on these 2 factors has become an essential area of research interest.

Some evidence suggests that dietary components, such as raspberries, can modulate inflammatory markers, including C-reactive protein (CRP) and IL-6 [15]. Elevated concentrations of these markers are often associated with increased risk of chronic diseases. Similarly, consistent blood glucose control is critical for preventing and managing diabetes. Emerging studies have suggested that raspberries may possess hypoglycemic properties, relying on their ability to activate cellular glucose transporters, inhibit α-glucosidase activity, and increase insulin sensitivity [[16], [17], [18]].

Despite individual studies exploring the impact of raspberry consumption on inflammatory markers and glycemic control, a comprehensive understanding of this relationship is lacking. Therefore, a systematic review and meta-analysis become crucial in synthesizing the available evidence, providing a more robust assessment of the impact of raspberry consumption on these 2 important measures. This systematic review and meta-analysis of randomized controlled trials aimed to investigate the effect of raspberry consumption on inflammatory markers and glycemic control, ultimately contributing to a deeper understanding of the potential health benefits of this fruit.

## Methods

This study was performed based on the PRISMA protocol for reporting systematic reviews and meta-analyses [19]. The protocol for conducting this systematic review is registered in the PROSPERO database with registration code: CRD42023477559. All the steps of this systematic review were carried out according to the protocol that was registered in the PROSPERO database.

### Search strategy

Two investigators (M.S.J. and M.C.) independently performed a systematic search in online databases, including PubMed, Scopus, and Web of Science, to find publications until December 2023 (18 December 2023). Additionally, we manually reviewed the reference lists of the included studies to identify relevant articles. The following keywords were used in the search: (“raspberry” OR “rubus occidentalis” OR “rubus idaeus” OR “rubus coreanus”) AND (“high sensitivity C-reactive protein” OR “hs-CRP” OR “CRP” OR “tumor necrosis factor” OR “TNF-α” OR “interleukin” OR “blood sugar” OR “fasting blood sugar” OR “fbs” OR “fasting plasma glucose” OR “insulin” OR “glycemia” OR “insulin resistance” OR “hemoglobin A1C” OR “A1C” OR “insulin sensitivity” OR “HOMA-IR” OR “homair”) AND (“randomized” OR “placebo” OR “clinical trials” OR “randomly” OR “trial” OR “randomized controlled trial” OR “RCT”).

### Eligibility criteria

The studies obtained from the initial search were independently screened using their title and abstracts, and if necessary, the full text of the articles was read to evaluate the eligible criteria by 2 researchers independently (M.S.J. and M.C.). Disagreements were discussed until a consensus was reached. This review did not include any language or time restrictions. Also, our review was not limited to studies published in specific journals.

Eligibility criteria for this systematic review included the following: *1*) only adult participants (over the age of 18 y), *2*) with an RCT design, *3*) intervention must be solely focused on raspberries and not combined with other nutrients, *4*) reported changes in glycemic control [fasting blood glucose (FBG), HbA1C, insulin, oral glucose tolerance test (OGTT), or HOMA-IR] or inflammation (hs-CRP, IL-6, or TNF-α) biomarker concentrations, and *5*) the studies must report the means and SDs of inflammation or glycemic markers before and after the intervention for both the intervention and control groups (or reported the mean changes and SD of outcomes for both groups).

#### Exclusion criteria

Studies with the following characteristics were excluded from this review: *1)* not having an appropriate control group; *2)* animal studies; *3)* studies conducted in a laboratory setting; *4)* intervention with raspberry consumption in combination with other nutrients; and *5)* observational studies including cohort, cross-sectional, and case–control; as well as literature reviews and review articles.

### Data extraction

Two investigators (M.S.J. and M.C.) performed data extraction independently. The following data were extracted from each eligible trial: first author's name, year of publication, country, study design, sample size, health status, number of participants and their mean age and mean BMI in each group, type of intervention, a daily dosage of intervention, duration of the study, and mean changes and SD of outcome levels for intervention and control groups. Disputed items were discussed until a consensus was reached.

### Quality assessment

The quality assessment of the studies was performed using the Cochrane quality assessment tool by 2 authors (M.S.J. and M.N.), independently to evaluate the risk of bias for each included study [20]. This tool assesses the risk of bias in 7 aspects: random sequence generation, allocation concealment, blinding personnel and participants, blinding of outcome assessment, incomplete outcome data, selective reporting, and other sources of bias. The risk of bias for each subclass was classified into 3 levels: low, unclear, and high. Disputes were resolved in consultation with the third author (A.S.).

### GRADE analyzing

The Grading of Recommendations Assessment, Development, and Evaluation (GRADE) Working Group guidelines were used to evaluate the quality of evidence for each assessed outcome.

### Statistical analysis

All statistical analyses were performed using Stata 17 software (Stata Corp). The overall size effect of raspberry was estimated for each outcome using weighted mean differences (WMD) and the SD of measures following the DerSimonian and Laird method [21]. If the mean changes were not reported, then the change was calculated by subtracting the initial values from the final values. If the SD of the change was not reported, then we estimated it using the following formula [22]: Change = square root [(SD_baseline_)^2^ + (SD_final_)^2^ − (2 × R × SD_baseline_ × SD_final_)]. The correlation coefficient of 0.9 was considered as *R* value that ranges between 0 and 1 [23]. SE, 95% CIs, and IQRs were converted to SDs using the approach of Hozo et al. [24]. Heterogeneity among included studies was evaluated by running Cochran's Q test and using the *I*-squared statistic (*I*^2^). *I*^2^ > 40% or *P* value < 0.05 was deemed as significant heterogeneity. Subgroup analyses were performed to find the source of heterogeneity based on the following criteria [25]: country (Korea and countries other than Korea), study design, baseline age (>35 compared with ≤35 y), baseline BMI (overweight and obesity), gender (both genders and females), trial duration (>8 compared with ≤8 wk), health status of participants, type of intervention (black raspberry and intervention other than black raspberry), and overall quality. Sensitivity analyses were carried out to check the effect of a particular study on the overall effect size of each outcome, using the leave-one-out method to determine what occurred after the elimination of 1 study at a time [26]. Publication bias among studies examining the effect of raspberry consumption on glycemic control or inflammatory markers was evaluated by Egger's regression and visual inspection of funnel plots [27]. In all the analyses performed, *P* values of <0.05 were considered statistically significant.

## Results

### Study selection

**FIGURE 1:**
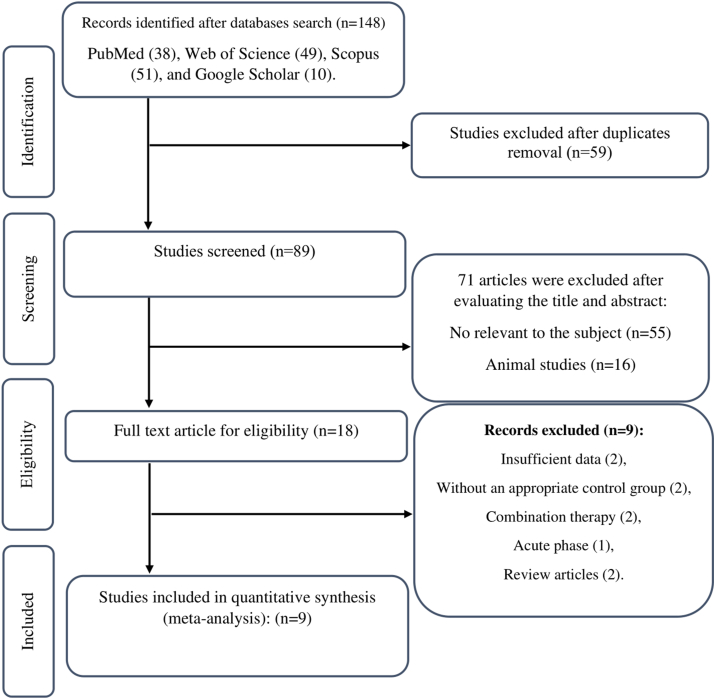
Flowchart of study selection for inclusion trials in the systematic review.

A total of 148 studies were obtained by searching the databases. After removing duplicates, 89 studies were screened using their titles and abstracts. The full text of 18 studies was read, and as a result, 9 studies that did not meet the inclusion criteria were excluded. Finally, 9 studies (12 arms) were included in this systematic review [11,15,[28], [29], [30], [31], [32], [33], [34]] (Figure 1).

### Study characteristic

Eligible studies were published from 2014 [11] to 2022 [34]. The study countries included Korea [11,[29], [30], [31], [32]], Iraq [28], Canada [33,34], and the United States [15]. The study conducted by Schell et al. [15] had a crossover design, whereas other studies had a parallel design. The sample size of the included studies varied from 22 [15] to 73 [11] participants. All studies were conducted in both genders, except in Mosah et al.’s [28], which was conducted in females only. Intervention populations included individuals with type 2 diabetes [15], metabolic syndrome (METS) [11,31], prediabetes [29], prehypertension [30], and at risk for METS [33,34]. Also one study was conducted on females with obesity [28], whereas the health state of the population was not reported in one study [32]. Among the effect sizes, the mean age of the participants ranged from 32.19 [33], to 59.3 y [29], and the mean BMI ranged from 24.4 [29] to 35.3 kg/m^2^ [15].

The raspberry interventions included raspberry ketones (*N* = 1) [28], red raspberry (*N* = 1) [15], or black raspberry (*N* = 5) [11,[29], [30], [31], [32]]. However, 2 studies did not report the type of raspberry intervention [33,34]. The duration of the intervention varied from 4 [15,35] to 12 wk [11,28,29,31]. The characteristics of the included studies are summarized in Table 1 [11,15,[28], [29], [30], [31], [32], [33], [34]].

**TABLE 1:** Characteristics of included studies in meta-analysis

Studies	Country	Study Design	Participant	Sample Size and Sex	Sample Size		Trial Duration (**wk**)	Mean Age		Means BMI		Intervention		Reported Outcomes
					IG	CG		IG	CG	IG	CG	Raspberries dose (mg/d)	Control group	
Jeong et al., 2014 [11]	Korea	Parallel, R, PC, DB	Patients with metabolic syndrome	73 B	38	35	12	58.0 ± 9.2	60.1 ± 9.5	26.3 ± 4.3	25.1 ± 4.0	Black raspberry (*Rubus occidentalis*) extract 750 mg/d	Placebo	CRP, IL-6, and TNF-α
Mosah et al. 2015 [28]	Iraq	Parallel, R, PC, SB	Females with obesity	38 F	20	18	12	31.75 ±5.58	32.72 ±7.00	35.41 ±3.34	34.83 ±2.99	Raspberry ketones 500 mg/d	without treatment	FBG
**An et al., 2016** [29] **(a)**	Korea	Parallel, R, PC, DB	Subjects with prediabetes	24 B	12	12	12	60.2 ± 8.6	58.4 ± 8.3	24.4 ± 2.3	24.4 ± 1.9	Low-dose black raspberry (*R. occidentalis*) 900 mg/d	Placebo	FBG, HbA1C, Insulin, GGT, HOMA-IR
**An et al., 2016** [29] **(b)**	Korea	Parallel, R, PC, DB	Subjects with prediabetes	27 B	15	12	12	58.4 ± 7.4	58.4 ± 8.3	25.0 ± 2.1	24.4 ± 1.9	High-dose black raspberry (R. *occidentalis*) 1800 mg/d	Placebo	FBG, HbA1C, Insulin, GGT, HOMA-IR
Jeong et al. 2016 [30] (a)	Korea	Parallel, R, PC, DB	Subjects with prehypertension	30 B	15	15	8	60.2±11.2	55.9±12.8	24.5±2.9	25.8±3.0	Moderate-dose black raspberry (*R. occidentalis*) dried powder extract 1500 mg/d	Placebo	CRP, IL-6, and TNF-α
Jeong et al., 2016 [30] (b)	Korea	Parallel, R, PC, DB	Subjects with prehypertension	30 B	15	15	8	55.5±12.3	55.9±12.8	23.5±2.4	25.8±3.0	High-dose black raspberry (R. *occidentalis*) dried powder extract 2500 mg/d	Placebo	CRP, IL-6, and TNF-α
Jeong et al., 2016 [31]	Korea	Parallel, R, PC, DB	Patients with Metabolic Syndrome	50 B	25	25	12	56.4 ±9.2	60.7± 10.4	25.9±4.6	24.7±3.9	Black raspberry (*R. occidentalis*) extract 750 mg/d	Placebo	CRP, IL-6, and TNF-α
Kim et al., 2017 [32] (a)	Korea	Parallel, R, PC, DB	Not reported	46 B	22	24	4	42 ± 8	45 ± 8	25 ± 2	25 ± 2	Freeze-dried powder black raspberry (*R. occidentalis*) 30,000 mg/d	Placebo	IL-6 and TNF-α
Kim et al., 2017 [32] (b)	Korea	Parallel, R, PC, DB	Not reported	45 B	21	24	4	46 ± 7	45 ± 8	25 ± 2	25 ± 2	Freeze-dried powder black raspberry (*R. coreanus*) 30,000 mg/d	Placebo	IL-6 and TNF-α
Schell et al., 2019 [15]	United States	Cross over, R, PC, DB	Adults with type 2 diabetes	22 B	22	22	4	54±19.69	54±19.69	35.3±9.38	35.3±9.38	Frozen red raspberries 250,000 mg/d	Maintained their usual diet	FBG, CRP, IL-6, and TNF-α
Franck et al., 2020 [33]	Canada	Parallel, R, PC, DB	Subjects at risk of metabolic syndrome	48 B	24	24	8	32.46±10.12	31.92±8.05	30.42±5.00	29.38±3.94	Frozen raspberries 280,000 mg/d	Maintained their health and food habits stable	FBG, HbA1C, Insulin, and CRP
Franck et al., 2022 [34]	Canada	Parallel, R, PC, DB	Participants with overweight or abdominal obesity, and with slight hyperinsulinemia or hypertriglyceridemia	24 B	13	11	8	32.6±10.5	34.0±9.5	29.2±3.9	32.8±5.7	Raspberries 280,000 mg/d	Maintained their usual diet	FBG, HbA1C, insulin, and CRP

Abbreviations: B, both genders; CG, control group; CO, controlled; CRP, C-reactive protein; DB, double-blinded; F, female; FBG, fasting blood sugar; Hb A1C, hemoglobin A1C; IG, intervention group; IL-6, interleukin 6; M, male; NR, not reported; OGTT, oral glucose tolerance test; PC, placebo-controlled; RA, randomized; SB, single-blinded; TNF-α, tumor necrosis factor-alfa.

(a) and (b) Indicate 2 treatment arms from one study.

The overall quality was considered good in all included studies, except in the 2 studies conducted by Mosah et al. [28] and Franck et al. [34], which were deemed fair. The details of quality assessment and the risk of bias in each subclass are summarized in Table 2 [11,15,[28], [29], [30], [31], [32], [33], [34]].

**TABLE 2:** Risk of bias assessment

Study	Random sequence generation	Allocation concealment	Selective reporting	Other sources of bias	Blinding (participants and personnel)	Blinding (outcome assessment)	Incomplete outcome data	General quality
Jeong et al., 2014 [11]	L	L	L	U	L	U	L	Good
Mosah et al., 2015 [28]	U	U	L	U	H	H	L	Fair
An et al., 2016 [29]	L	L	L	U	L	U	L	Good
Jeong et al., 2016 [30]	L	U	L	U	L	U	L	Good
Jeong et al., 2016 [31]	L	L	L	U	L	U	L	Good
Kim et al., 2017 [32]	L	L	H	L	L	U	L	Good
Schell et al., 2019 [15]	U	L	L	L	H	U	L	Good
Franck et al., 2020 [33]	L	L	L	L	H	U	L	Good
Franck et al., 2022 [34]	U	U	L	U	H	U	L	Fair

Abbreviations: L, low risk of bias; H, high risk of bias; U, unclear risk of bias.

General good quality: low risk >2 items; general fair quality: low risk =2 items; general poor quality: low risk <2 items.

### Meta-analysis

#### Effect of raspberry consumption on FBG

**FIGURE 2:**
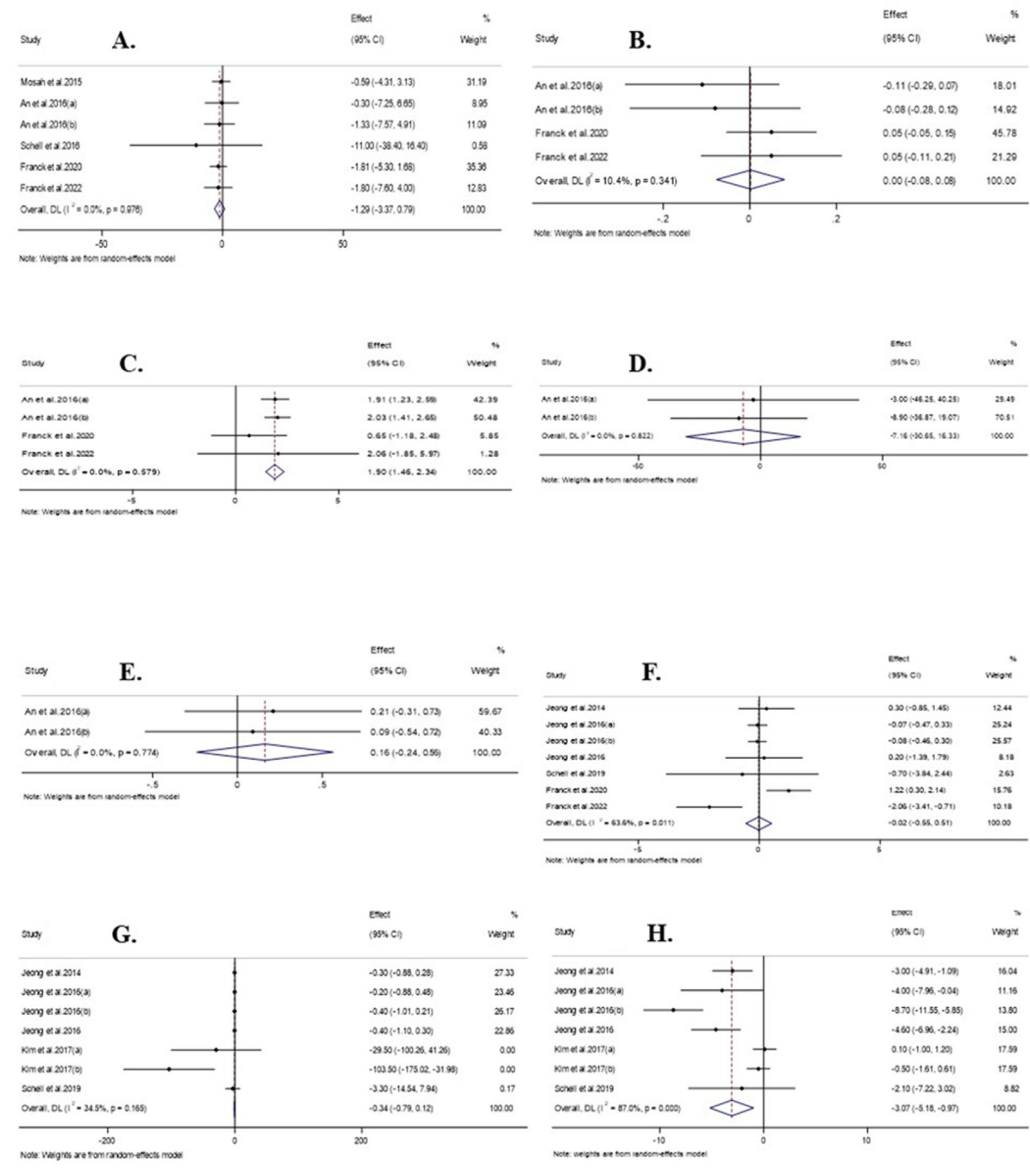
Forest plot detailing weighted mean difference and 95% confidence intervals (CIs) for the effect of raspberry intake on (A) FBG (mg/dL); (B) HbA1C (%); (C) insulin (μU/mL); (D) GGT (mg/dL); (E) HOMA-IR; (F) CRP (mg/L; (G) IL-6 (pg/mL); and (H) TNF-α (pg/mL). Abbreviations: CRP, C-reactive protein; FBG, fasting blood sugar; Hb A1C, hemoglobin A1C; IL-6, IL-6; TNF-α, tumor necrosis factor-alfa.

The combination of 6 effect sizes showed that raspberry consumption had no significant effect on FBG concentrations compared with the control groups (WMD: −1.29 mg/dL; 95%CI: −3.37, 0.78; *P* = 0.22) (Figure 2A). Also, the included studies had no significant heterogeneity (*I*^2^ = 0.0%; *P* = 0.97). Subgroup analysis, conducted to investigate the effect of raspberry consumption on FBG concentrations in each of the predefined subgroups, demonstrated that raspberry consumption did not significantly change FBG concentrations in any of the predetermined subgroups (Table 3).

**TABLE 3:** Subgroup analyses of raspberry consumption on glycemic control and inflammation markers in adults

	No.	WMD (95%CI)	*P* value	Heterogeneity		
				*P* heterogeneity	*I*^2^ (%)	*P* between subgroups
Subgroup analyses of raspberry consumption on FBG (mg/dL)						
Overall effect	6	−1.29 (−3.37, 0.78)	0.22	0.97	0.0	
Country						
Korea	2	−0.87 (−5.51, 3.77)	0.71	0.82	0.0	0.84
Non-Korea	4	−1.39 (−3.72, 0.92)	0.23	0.86	0.0	
Trial duration (wk)						
≤8	3	−1.91 (−4.89, 1.06)	0.20	0.80	0.0	0.56
>8	3	−0.70 (−3.60, 2.20)	0.63	0.97	0.0	
Raspberry type						
Black raspberry	2	−0.87 (−5.51, 3.77)	0.71	0.82	0.0	0.84
Non-black raspberry	4	−1.39 (−3.72, 0.92)	0.23	0.86	0.0	
Health status						
T2DM	2	−1.15 (−5.72, 3.42)	0.62	0.75	0.0	0.88
At risk of METS	2	−1.80 (−4.80, 1.18)	0.23	0.99	0.0	
Others	1	−0.59 (−4.31, 3.13)	0.75			
Age (y)						
≤35	3	−1.32 (−3.66, 1.00)	0.26	0.88	0.0	0.94
>35	3	−−1.15 (−5.72, 3.42)	0.62	0.75	0.0	
Gender						
Both genders	5	−1.61 (−4.11, 0.89)	0.20	0.96	0.0	0.65
Female	1	−0.59 (−4.31, 3.13)	0.75			
Baseline BMI						
Overweight	2	−0.87 (−5.51, 3.77)	0.71	0.82	0.0	0.84
Obesity	4	−1.39 (−3.72, 0.92)	0.23	0.86	0.0	
Overall quality						
Good	4	−1.56 (−4.34, 1.20)	0.26	0.89	0.0	0.77
Fair	2	−0.94 (−4.07, 2.18)	0.55	0.73	0.0	
Study design						
Parallel, R, PC, DB	4	−1.53 (−4.04, 0.98)	0.23	0.98	0.0	0.72
Cross over, R, PC, DB	1	−11.00 (−38.39, 16.39)	0.43			
Parallel, R, PC, SB	1	−0.59 (−4.31, 3.13)	0.75			
Subgroup analyses of raspberry consumption on A1C (%)						
Overall effect	4	0.002 (−0.07, 0.08)	0.96	0.34	10.4	
Country						
Korea	2	−0.09 (−0.22, 0.03)	0.15	0.82	0.0	0.06
Non-Korea	2	0.05 (−0.03, 0.13)	0.26	1.000		
Trial duration (wk)						
≤8	2	0.05 (−0.03, 0.13)	0.26	1.000		0.06
>8	2	−0.09 (−0.22, 0.03)	0.15	0.82	0.0	
Raspberry type						
Black raspberry	2	−0.09 (−0.22, 0.03)	0.15	0.82	0.0	0.06
Non–black raspberry	2	0.05 (−0.03, 0.13)	0.26	1.000		
Health status						
Prediabetes	2	−0.09 (−0.22, 0.03)	0.15	0.82	0.0	0.06
At risk of METS	2	0.05 (−0.03, 0.13)	0.26			
Age (y)						
≤35	2	0.05 (−0.03, 0.13)	0.26	1.000		0.06
>35	2	−0.09 (−0.22, 0.03)	0.15	0.82	0.0	
Baseline BMI						
Overweight	2	−0.09 (−0.22, 0.03)	0.15	0.82	0.0	0.06
Obesity	2	0.05 (−0.03, 0.13)	0.26	1.000		
Overall quality						
Good	3	−0.02 (−0.12, 0.08)	0.68	0.22	32.9	0.46
Fair	1	0.05 (−0.11, 0.21)	0.54			
Subgroup analyses of raspberry consumption on insulin (μU/mL)						
Overall effect	4	1.89 (1.45, 2.34)	<0.001	0.57	0.0	
Country						
Korea	2	1.97 (1.51, 2.43)	<0.001	0.79	0.0	0.22
Non-Korea	2	0.90 (−0.75, 2.56)	0.28	0.52	0.0	
Trial duration (week)						
≤8	2	0.90 (−0.75, 2.56)	0.28	0.52	0.0	0.22
>8	2	1.97 (1.51, 2.43)	<0.001	0.79	0.0	
Raspberry type						
Black raspberry	2	1.97 (1.51, 2.43)	<0.001	0.79	0.0	0.22
Non–black raspberry	2	0.90 (−0.75, 2.56)	0.28	0.52	0.0	
Health status						
Prediabetes	2	1.97(1.51,2.43)	<0.001	0.79	0.0	0.22
At risk of METS	2	0.90(−0.75, 2.56)	0.28	0.52	0.0	
Age (y)						
≤35	2	0.90(−0.75, 2.56)	0.28	0.52	0.0	0.22
>35	2	1.97(1.51, 2.43)	<0.001	0.79	0.0	
Baseline BMI						
Overweight	2	1.97 (1.51, 2.43)	<0.001	0.79	0.0	0.22
Obesity	2	0.90 (−0.75, 2.56)	0.28	0.52	0.0	
Overall quality						
Good	3	1.89 (1.45, 2.34)	<0.001	0.37	0.0	0.93
Fair	1	2.06 (−1.85, 5.97)	0.30			
Subgroup analyses of raspberry consumption on HOMA-IR						
Overall effect	2	0.16 (−0.24, 0.56)	0.43	0.77	0.0	
Subgroup analyses of raspberry consumption on OGTT (mg/dL)						
Overall effect	2	−7.16 (−30.64, 16.32)	0.55	0.82	0.0	
Subgroup analyses of raspberry consumption on CRP (mg/L)						
Overall effect	7	−0.02 (−0.55, 0.51)	0.94	0.01	63.6	
Country						
Korea	4	−0.04 (−0.31,0.21)	0.71	0.92	0.0	0.75
Non-Korea	3	−0.45 (−2.95, 2.04)	0.72	<0.001	87.3	
Trial duration (wk)						
≤8	5	−0.11 (−0.78, 0.55)	0.74	0.003	75.1	0.51
>8	2	0.26 (−0.66, 1.19)	0.57	0.92	0.0	
Raspberry type						
Black raspberry	4	−0.04 (−0.31,0.21)	0.71	0.92	0.0	0.75
Non–black raspberry	3	−0.45 (−2.95, 2.04)	0.72	<0.001	87.3	
Health status						
METS	2	0.26 (−0.66, 1.19(	0.57	0.92	0.0	0.87
Prehypertension	2	−0.07 (−0.34, 0.19)	0.58	0.97	0.0	
T2DM	1	−0.70 (−3.84, 2.44)	0.66			
At risk of METS	2	−0.38 (−3.59, 2.83)	0.81	<0.001	93.5	
Age (y)						
≤35	2	−0.02 (−0.55, 0.51)	0.81	<0.001	93.5	0.84
>35	5	−0.05 (−0.31, 0.20)	0.69	0.95	0.0	
Baseline BMI						
Overweight	1	−0.08 (−0.45, 0.29)	0.67			0.94
Obesity	6	−0.04 (−0.86, 0.76)	0.90	0.006	69.4	
Overall quality						
Good	6	0.13 (−0.24, 0.51)	0.48	0.18	33.4	0.002
Fair	1	−2.06 (−3.41, −0.70)	0.003			
Study design						
Parallel, R, PC, DB	6	−0.004 (−0.56, 0.55)	0.98	0.006	69.3	0.66
Cross over, R, PC, DB	1	−0.70) −3.84, 2.44(	0.66			
Subgroup analyses of raspberry consumption on IL-6 (pg/mL)						
Overall effect	7	−0.33 (−0.79, 0.12)	0.15	0.16	34.5	
Country						
Korea	6	−0.33 (−0.81, 0.15)	0.18	0.11	43.7	0.60
Non-Korea	1	−3.30 (−14.53, 7.93)	0.56			
Trial duration (wk)						
≤8	5	−0.36 (−1.48,0.75)	0.52	0.05	56.1	0.97
>8	2	−0.34 (−0.78, 0.10)	0.13	0.82	0.0	
Raspberry type						
Black raspberry	6	−0.33 (−0.81, 0.153)	0.18	0.11	43.7	0.60
Non-black raspberry	1	−3.30 (−14.53, 7.93)	0.56			
Health status						
METS	2	−0.34 (−0.78, 0.10)	0.13	0.82	0.0	0.32
Prehypertension	2	−0.31 (−0.76, 0.14)	0.17	0.66	0.0	
T2DM	1	−3.30 (−14.53, 7.93)	0.56			
Others	2	−66.31 (−138.82, 6.20)	0.07	0.14	51.9	
Baseline BMI						
Overweight	3	−37.00 (−98.11, 24.09)	0.23	0.01	76.8	0.23
Obesity	4	−0.30 (−0.67, 0.07)	0.11	0.93	0.0	
Study design						
Parallel, R, PC, DB	6	−0.33 (−0.81, 0.15)	0.18	0.11	43.7	0.60
Cross over, R, PC, DB	1	−3.30 (−14.53, 7.93)	0.56			
Subgroup analyses of raspberry consumption on TNF-α (pg/mL)						
Overall effect	7	−3.07 (−5.17,−0.97)	0.004	<0.001	87.0	
Country						
Korea	6	−3.18 (−5.43, −0.92)	0.006	<0.001	89.2	0.70
Non-Korea	1	−2.10 (−7.22, 3.02)	0.42			
Trial duration (wk)						
≤8	5	−2.79 (−5.46, −0.12)	0.04	<0.001	88.6	0.58
>8	2	−3.64 (−5.18, −2.10)	<0.001	0.30	6.2	
Raspberry type						
Black raspberry	6	−3.18 (−5.43, −0.92)	0.006	<0.001	89.2	0.70
Non–black raspberry	1	−2.10 (−7.22, 3.02)	0.42			
Health status						
METS	2	−3.00 (−4.912, −1.08)	<0.001	0.30	6.2	<0.001
Prehypertension	2	−6.56 (−11.14, −1.97)	0.005	0.05	71.9	
T2DM	1	−2.10 (−7.22, 3.02)	0.42			
Others	2	−0.20 (−0.98, 0.58)	0.61	0.45	0.0	
Baseline BMI (kg/m^2^)						
Overweight	3	−2.67 (−6.03, 0.68)	0.11	<0.001	93.8	0.62
Obesity	4	−3.57 (−4.91, −2.22)	<0.001	0.69	0.0	
Study design						
Parallel, R, PC, DB	6	−3.18 (−5.43, −0.92)	0.006	<0.001	89.2	0.70
Cross over, R, PC, DB	1	−2.10 (−7.22, 3.023)	0.42			

Abbreviations: BMI, body mass index; CI, confidence interval; CRP, C-reactive protein; FBG, fasting blood sugar; Hb A1C, hemoglobin A1C; IL-6, interleukin 6; METS, metabolic syndrome; OGTT, oral glucose tolerance test; parallel, R, PC, DB, parallel, randomized placebo-controlled double-blind; parallel, R, PC, SB, parallel, randomized placebo-controlled single-blind; T2DM, type 2 diabetes mellitus; WMD, weighted mean differences.

In non-Korean populations 25 ≤ BMI <30 kg/m^2^ identified as overweight and 30 kg/m^2^ ≤ BMI identified as obesity.

In Korean populations 23 ≤ BMI <25 kg/m^2^ identified as overweight and 25 kg/m^2^ ≤ BMI identified as obesity.

#### Effect of raspberry consumption on HbA1C

A meta-analysis of 4 effect sizes revealed that raspberry consumption did not significantly affect HbA1C concentrations compared with control groups (WMD: 0.002%; 95%CI: −0.07, 0.08); *P* = 0.96) (Figure 2B). Furthermore, no significant heterogeneity was detected among the included studies (*I*^2^ = 10.4%; *P* = 0.34). No significant change in HbA1C concentrations followed by raspberry consumption was reported in any predetermined subgroups (Table 3).

#### Effect of raspberry consumption on insulin

Pooling of 4 effect sizes showed that raspberry consumption significantly increased insulin concentrations compared with the control groups (WMD: 1.89 μU/mL; 95%CI: 1.45, 2.34; *P* < 0.001) (Figure 2C). Also, no significant heterogeneity was observed among the included studies (*I*^2^ = 0.0%; *P* = 0.57). Subgroup analysis demonstrated that raspberry consumption significantly increased the insulin concentrations in studies conducted in Korea, with a duration of >8 wk, intervened with black raspberry, or were conducted on individuals with overweight, prediabetes, or age >35 y (Table 3).

#### Effect of raspberry consumption on OGTT

The combination of 2 effect sizes showed that raspberry consumption had no significant effect on OGTT concentrations compared with control groups (WMD: −7.16 mg/dL; 95% CI: −30.64, 16.32; *P* = 0.55; *I*^2^ = 0.0%; *P* = 0.82) (Figure 2D).

#### Effect of raspberry consumption on HOMA-IR

The combination of 2 effect sizes demonstrated that raspberry consumption did not significantly change HOMA-IR compared with the control groups (WMD: 0.16; 95% CI: −0.24, 0.56; *P* = 0.43; *I*^2^ = 0.0%; *P* = 0.77) (Figure 2E).

#### Effect of raspberry consumption on CRP

Pooling 7 effect sizes showed that raspberry consumption did not significantly change CRP concentrations compared with control groups (WMD: −0.02 mg/L; 95%CI: −0.55, 0.51); *P* = 0.94) (Figure 2F). At the same time, there was significant heterogeneity among the included studies (*I*^2^ = 63.6%; *P* = 0.01). Subgroup analysis demonstrated that raspberry intake led to a significant decrease in CRP concentrations in the studies with fair overall quality (Table 3).

#### Effect of raspberry consumption on IL-6

Performing meta-analysis on 7 effect sizes demonstrated that raspberry consumption did not significantly affect IL-6 concentrations compared with the control groups (WMD, −0.33 pg/mL; 95%CI: −0.79, 0.12; *P* = 0.15) (Figure 2G). Also, no significant heterogeneity was observed among the included studies (*I*^2^ = 34.5%; *P* = 0.16). Subgroup analysis showed that raspberry consumption did not significantly affect IL-6 concentrations in any of the determined subgroups (Table 3).

#### Effect of raspberry consumption on TNF-α

Pooling 7 effect sizes revealed that raspberry consumption led to a significant decrease in TNF-α concentrations compared with the control groups (WMD: −3.07 pg/mL; 95%CI: −5.17, −0.97; *P* = 0.004) (Figure 2H). Although significant heterogeneity was observed among the included studies (*I*^2^ = 87.0%; *P* < 0.001). Subgroup analysis showed that raspberry intake in studies with a crossover design, interventions with nonblack raspberry, or trials were conducted on individuals with overweight or diabetes or in countries other than Korea, leading to nonsignificant changes in TNF-α concentrations (Table 3).

### Sensitivity analysis

The sensitivity analysis showed that the overall effect size of raspberry consumption on IL-6 concentrations after omitting Kim et al.’s [32] treatment arm (b) (WMD: −0.32 pg/mL; 95%CI: −0.64, −0.01) [32], significantly changed. However, the pooled effect size of other outcomes, including FBG, HbA1C, insulin, CRP, and TNF-α, was not significantly affected by the presence of only one specific trial.

### Publication bias

**FIGURE 3:**
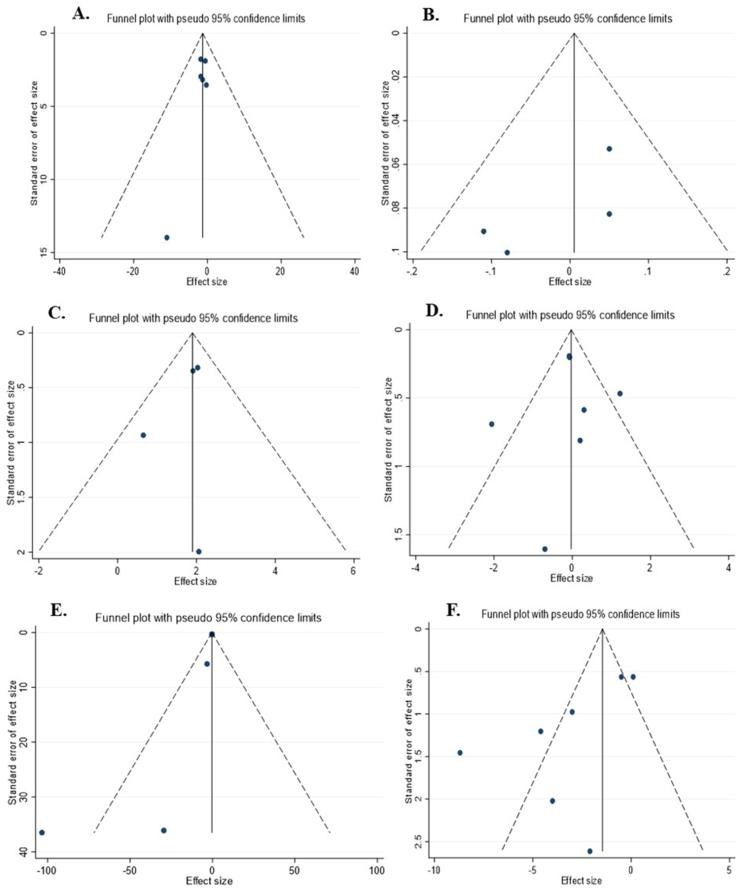
Funnel plots for the effect of raspberry intake on (A) FBG (mg/dL); (B) HbA1C (%); (C) insulin (μU/mL); (D) CRP (mg/L); (E) IL-6 (pg/mL); and (F) TNF-α (pg/mL). Abbreviations: CRP, C-reactive protein; FBG, fasting blood sugar; Hb A1C, hemoglobin A1C; IL-6, interleukin 6.

Egger's regression and visual interpretation of the funnel plot showed a significant publication bias among the included studies investigating the effect of raspberry consumption on IL-6 concentrations (*P*_Egger_ = 0.03). Otherwise, no significant publication bias was reported among the included studies for other outcomes (Figure 3A–F).

### GRADE analysis

The quality of the evidence included in this meta-analysis was evaluated based on the GRADE framework. The GRADE analysis determined the quality of the evidence investigating the effect of raspberry consumption on insulin concentrations as high. Furthermore, the quality of evidence for FBG and HbA1C was moderate, whereas the quality of the evidence for CRP, IL-6, and TNF-α was downgraded to low (GRADE profile for glycemic control and inflammation markers are provided in Table 4).

**TABLE 4:** GRADE profile of raspberry consumption for glycemic control and inflammation markers

Quality assessment						Quality of evidence
Outcomes	Risk of bias	Inconsistency	Indirectness	Imprecision	Publication bias	
FBG (mg/dL)	No serious limitations	No serious limitations	No serious limitations	Serious limitations^1^	No serious limitations	⊕⊕⊕◯ Moderate
HbA1C (%)	No serious limitations	No serious limitations	No serious limitations	Serious limitations^1^	No serious limitations	⊕⊕⊕◯ Moderate
Insulin (μU/mL)	No serious limitations	No serious limitations	No serious limitations	No serious limitations	No serious limitations	⊕⊕⊕⊕ High
CRP (mg/L)	No serious limitations	Serious limitations^2^	No serious limitations	Serious limitations^1^	No serious limitations	⊕⊕◯◯ Low
IL-6 (pg/mL)	No serious limitations	No serious limitations	No serious limitations	Serious limitations^1^	Serious limitations^3^	⊕⊕◯◯ Low
TNF-α (pg/mL)	No serious limitations	Very serious limitations^4^	No serious limitations	No serious limitations	No serious limitations	⊕⊕◯◯ Low

Abbreviations: CI, confidence interval; CRP, C-reactive protein; FBG, fasting blood sugar; Hb A1C, hemoglobin A1C; IL-6, interleukin 6; WMD, weighted mean differences.

1 There is no evidence of significant effects of raspberry intake.

2 There is high heterogeneity (*I*^2^> 40%).

3 There is a significant publication bias based on Egger’s test.

4 There is very high heterogeneity (*I*^2^> 75%).

## Discussion

The results of the present meta-analysis indicated that raspberry consumption could have significant effects on insulin and TNF-α concentrations. However, the findings did not support any favorable effects on serum FBG, HbA1C, HOMA-IR, IL-6, and CRP concentrations. Also, subgroup analysis showed no significant changes in FBG, HbA1C, and IL-6 concentrations.

However, the results of some previous studies indicated that raspberries have favorable properties, including improvement of lipid metabolism, antioxidant and anti-inflammatory activity, and signaling regulatory effects through the bioactive components such as polyphenols and anthocyanins, which can reduce the risk of chronic metabolic diseases [[36], [37], [38]].

The subgroup analysis revealed that raspberries generally increase insulin concentrations, and this effect depends on the raspberry type, trial duration, and characteristics of the participants (health status, age, and baseline BMI).

The significant enhancing effect of raspberry consumption on insulin concentrations was observed in the studies that were conducted in Korea, with good overall quality or trials with duration of >8 wk, or intervened with black raspberry as well as trials conducted on individuals with overweight, prediabetes, or age > 35 y. The potential mechanism of raising insulin secretion followed by raspberry extract consumption may be related to its bioactive components. It has been demonstrated that anthocyanins and anthocyanidins can elevate insulin secretion from pancreatic β-cells [39]. In addition, in one study in the Korean population, the investigators observed that pancreatic β-cells activity was improved after 12 wk of intervention with black raspberry extract [29]. However, this finding was in conflict with the results of some previous studies that reported the lowering effect of anthocyanins on insulin concentrations [40]. Also, this difference can be attributed to higher amounts of carbohydrate intake when consuming raspberries than when taking anthocyanin supplements, which can stimulate more insulin secretion.

The subgroup analysis based on the health status of participants revealed that insulin concentrations significantly increased after raspberry consumption in the individuals with prediabetes. Also, studies reported that raspberry's favorable insulin signaling effects were observed in individuals with prediabetes [15,41]. This significant effect was not noted in people at risk for METS. More studies on individuals with METS and diabetes need to clarify whether raspberry has insulinotropic effect or not. Our findings showed that raspberry increased insulin concentrations in studies conducted on participants with overweight. The experimental study on mice reported that fat accumulation in muscle mass attenuates insulin signaling by decreasing AMP-activated protein kinase activity, which is a crucial factor in glucose metabolism and related to diabetes and metabolic disorders [42]. The increased insulin concentrations, along with the nonsignificant changes in other glycemic control markers following raspberry consumption, led to the fact that these findings should be interpreted with caution, and definitive conclusions should be postponed until RCTs with a larger sample size are performed in the future.

Our meta-analysis did not indicate any significant effects on FBG, HbA1C, OGTT, and HOMA-IR with raspberries, whereas several studies have reported favorable changes in glycemic control markers after raspberry consumption [15,29,42].

Our meta-analysis revealed that raspberry had a significant lowering effect on TNF-α concentrations, whereas its effects on CRP and IL-6 were not significant. This could be related to the role of TNF-α as an earlier indicator of inflammation compared with CRP and IL-6 [43,44].

Furthermore, subgroup analysis showed that the efficacy of raspberry consumption on TNF-α was more perceptible in the studies that intervened with black raspberry, or trials conducted on the Korean population or individuals with obesity, METS, or prehypertension. However, the heterogeneity of the included studies was high. The anti-inflammatory and metabolic effects of raspberry have presented either with raspberry fruit or its purified extraction [33,[45], [46], [47], [48]]. However, some studies demonstrated its immunomodulatory properties attributed to phenolic components. These results are consistent with those detected with black raspberry [11,31].

Lowering oxidative stress is one way raspberries may be able to lower TNF-α [11]. The role of oxidative stress and oxidized low-density lipoprotein in METS has been reported in previous studies [49]. Infiltration of LDL into subendothelial space (where plasma antioxidants are not present) leads LDL to become oxidized by reactive oxygen species derived from leukocytes [50]. Oxidized LDL stimulates macrophages to form foam cells. Activation of macrophages increases the secretion of inflammatory cytokines, including IL-6, IL-1β, and TNF-α [11,51]. Excessive concentrations of cytokines due to tissue damages. Reduced serum TNF-α and IL-6 with black raspberry by decreased uptake of LDL cholesterol have been observed in previous findings [11]. In addition, the anti-inflammatory colon-derived catabolites of raspberry, including 3,4-dihydroxyphenylacetic acid, dihydroferulic acid, and dihydrocaffeic acid, were able to decrease the expression of TNF-α in vitro. Included studies have mentioned no toxicity of raspberry consumption in doses used. Generally, it might be safe and have no serious adverse effects; however, few investigations have been performed to evaluate possible side effects and safety of long-term and high dosages of raspberry intakes.

### Limitations

The review's limitations included a small number of included studies and sample sizes, nonuniformity in the type and form of raspberries consumed by participants, significant heterogeneity among included studies to investigate the effect of raspberry consumption on CRP and TNF-α concentrations, and low-quality evidence regarding TNF-α, IL-6, and CRP.

### Conclusions

To our knowledge, the present meta-analysis is the first comprehensive evaluation of the efficacy of raspberry in glycemic control and inflammatory markers. Our findings suggested that raspberry consumption can significantly reduce TNF-α and increase insulin concentrations. According to the insufficient and heterogenous studies, more investigations are necessary to confirm raspberry's possible effectiveness and usefulness for metabolic and inflammatory markers.

## Author contributions

The authors’ responsibility were as follows –; AS: conceptualization, project administration and writing – review and editing; MSJ: conceptualization, screening, data extraction, quality assessment, and writing – original draft; MN: quality assessment and writing – original draft; MC: screening, data extraction, and writing – original draft; AS and TJ: contributed to the revision and editing of the first draft; MSJ and AS: contributed in conception, data collection, and manuscript revising. MSJ, MN, and MC: contributed in conception, data collection, screening, data extraction, and manuscript drafting and all authors: read and approved the final version of the manuscript.

## Conflict of interest

The authors report no conflicts of interest.

## Funding

The authors reported no funding received for this study.

## Data availability

Data will be made available on request. All data generated or analyzed during this study are included in this published article.
